# Death from the Inhalation of Nitrous Oxide Gas

**Published:** 1872-05

**Authors:** W. J. Purcell

**Affiliations:** New York.


### SELECTED ARTICLES.

---

### ARTICLE VI.

### *Death from the Inhalation of Nitrous Oxide Gas.*

BY W. J. PURCELL, M. D., NEW YORK.

On Wednesday, March 20th, Mrs. Ann O'Shaughnessy visited the office of a dentist named Newbrough, No. 128 West 34th street, New York city, for the purpose of having some teeth extracted, she having suffered considerably for some time previously from toothache. Although desirous of taking the gas, she yet expressed a great dread of it, and it was with considerable difficulty that she was persuaded to submit. One bagful containing about four gallons was administered, but not without great trouble from the struggles and resistance of the patient, who appeared to labor under severe nervous irritability. This not being sufficient to produce anæsthesia, a second bagful was given, and after a few inhalations the dentist proceeded to extract the teeth. About four were removed, when the patient's head was observed to fall over on her left shoulder, and she was discovered to be insensible. Efforts were made to revive her, but in vain. It was found that she had ceased to breathe, and that no pulsation of the heart could be discerned. A physician was hastily summoned, and the galvanic battery and other means and appliances used, but without success. The woman was dead. The remain were removed to the late residence of the deceased, and an autopsy made, which revealed a slight congestion of the lungs.

An inquest was held before Coroner Herman, and a jury composed of prominent medical men. Several physicians who were present at the *post mortem* examination gave as the result, that death was caused by nervous shock ; there was slight congestion of the lungs.

The following embodies the most important part of the testimony, as it throws considerable light on the different methods of administering the gas, its modes of manufacture, etc., etc.

Dr. Buchanan, of 355 West Thirtieth St., testified that he was present at the *post mortem* examination made on the body of Mrs. O'Shaughnessy, at the residence of the deceased. He corroborated the testimony of the physicians previously examined as to the result of the autopsy. He said that in his opinion the cause of death was a nervous shock, consequent upon having the teeth extracted. He believed the disease had an incipient stage of pneumonia. In addition to the shock he thought that the inhaling of the gas might be a predisposing cause. The witness said that he gives nitrous oxide in cases of asthma ; he was careful when there was anything the matter with the heart ; he thought a physical examination of the patient necessary before giving the gas. When a patient dreaded the inhalation of the gas he would not give it for fear of producing a shock. He never saw a case illustrative of this idea, but he thought the preseut a very appropriate one. He thought that if all the proceedings had ended when the deceased had expressed a fear of taking the gas, nothing would have occurred ; that is, had not the teeth been drawn. The gas ought to be taken through a mouth-piece, provided with an outlet valve through which the expired gas could escape into the atmosphere. He could not say whether the apparatus used by deceased had such a mouth-piece. He could not say whether the quantity of gas taken by the deceased had anything to do with her death. He deprecated the custom of holding the nose, and breathing into the bag without means of escape for the impure gases. This last statement was in reply to a question from Dr. Rogers.

Hermann Endemann, chemist to the Board of Health of New York, testified that at the request of Coroner Hermann he examined the gas used by Dr. Newbrough and the material used in the manufacture of the same. The nitrate of ammonia used in the manufacture of the gas was not quite pure, but was as good as is usually provided. The apparatus used by Dr. Newbrough is capable of producing pure gas provided that it is administered within three hours after it is made. He made inquiries of Dr. Newbrough how often he made the gas, and he said every day. At a second visit paid by the witness to Dr. Newbrough's place the latter said that the gas he had administered to the deceased had been made two days before he gave it; he did not examine this gas, as it had all escaped. The gas he made with Dr. Newbrough's apparatus himself was pure; he inhaled it himself. The bag used by the deceased contained four gallons, with a mouth-piece, out of which the patient directly inhales the gas; the carbonic acid of exhalation thus enters the bag again. The witness thought that all mouth-pieces should have a safety-valve, through which the carbonic acid could escape. He thought that the deceased partly inhaled the nitrous oxide; the face becomes gradually livid under its influenee about fifty seconds after inhaling it; the pupils become distended, if the pulse is weak at first it generally becomes more rapid. The patient comes out of the influence in two or three minutes so as to be conscious, but the dizziness continues five minutes. The only experience the witness had of any evil results from inhaling the gas was in his assistant, who took it for the purpose of having a tooth extracted; next day he did not feel well in his lungs, feeling bad for about a week after. A physician told him that his lungs were congested; three months afterward he died of consumption. If the impurities of the gas be not removed he considered it harmful. He has noticed that gas has different effects upon individuals, producing depressing effects on some. He considered that the apparatus, with the same material used as he had examined, is capable of generating gas sufficiently pure, provided it is used three hours after being made.

Mary Mulligan, residing at 301 East Fifty-fifth street, testified that she is a dress-maker, employed at McCleary's in Broadway. Last summer the deceased worked at the same place with her; she was in good health, and never knew her to have fainting fits.

Mr. E. P. Hoyt, of 34 West Twenty-sixth street, said that he had been a dentist for fifteen years. He had used nitrous oxide gas extensively years ago, until he became alarmed, and discontinued its use on account of its tendency to produce asphyxia. About the year 1863 he almost discontinued its use, until he invented a different method of inhaling it, by allowing an escape of the carbonic acid gas. He was pressed very much by his patients to employ the gas, but he did not like to administer it without a physician being present. He is not entirely satisfied that it is harmless when given to produce anæsthesia. He would not give it in a case of heart disease or palpitation of the heart, or disease of the lungs or brain. The gas, he finds, acts as a stimulant; a little of it acts as a tonic.

Deputy Coroner, John Beach testified to having made a *post mortem* examination. His testimony agreed substantially with that of the other physicians.

THE VERDICT.

The jury find that the diseased, Ann O'Shaughnessy came to her death by asphyxia or apnœa, as evidenced by the symptoms manifested by the patient before death, and the conditions found at the *post mortem* examination; the asphyxia, in our opinion, being induced by the inhalation of gas administered.

The jury further agree in the opinion that any other than the prescribed method of preparation of nitrous oxide gas as to temperature for generation, and appropriate washing bottles, is to be regarded as a careless method of manufacture.

In this particular we regard the apparatus used by Dr. Newbrough as imperfect. Furthermore, we regard the administration of nitrous oxide gas as reckless where each administration is an experiment upon the patient, which it must be when there is not a positive certainty as to the chemical purity of the gas, and ignorance in the administrator of the physical condition of the patient and the physiological effect manifested by anæsthesia.

Furthermore, we regard it as necessary for safety that an administrator of nitrous oxide gas should have a knowledge of the application of restorative agents adapted to alarming conditions in anæsthesia, and be always supplied with such agents.—*Med. and Surg. Reporter.*

---

